# Identification of disease states associated with coagulopathy in trauma

**DOI:** 10.1186/s12911-016-0360-x

**Published:** 2016-09-22

**Authors:** Yuanyang Zhang, Tie Bo Wu, Bernie J. Daigle, Mitchell Cohen, Linda Petzold

**Affiliations:** 1 Department of Computer Science, University of California, Santa Barbara, USA; 2 Department of Mechanical Engineering, University of California, Santa Barbara, USA; 3 Departments of Biological Sciences and Computer Science, University of Memphis, Memphis, USA; 4 Department of Surgery, University of California, San Francisco, USA; 5 Department of Computer Science, University of California, Santa Barbara, USA

**Keywords:** Trauma, Coagulopathy, State identification, Hidden Markov model, Missing data

## Abstract

**Background:**

Trauma is the leading cause of death between the ages of 1 to 44 in the United States. Blood loss is the primary cause of these deaths. The discrimination of states through which patients transition would be helpful in understanding the disease process, and in identification of critical states and appropriate interventions. Even though these states are strongly associated with patients’ blood composition data, there has not been a way to directly identify them. Statistical tools such as hidden Markov models can be used to infer the discrete latent states from the blood composition data.

**Methods:**

We applied a hidden Markov model to time-series multivariate patient measurements from the UCSF/ San Francisco General Hospital and Trauma Center. Ten blood factor related measurements were used to identify the model: factors II, V, VII, VIII, IX, X, antithrombin III, protein C, prothrombin time and partial thromboplastin time. Missing data in the time-series dataset was considered in the hidden Markov model. The number of states was determined by minimizing the Bayesian information criterion across different numbers of states.

**Results:**

After preprocessing, 1090 patients with a total number of 2176 time point measurements were included in the analysis. The hidden Markov model identified 6 disease states and 3 stages. We analyzed their relationships to the blood composition data and the coagulation cascade. The states are very indicative of the disease progression status of patients.

**Conclusions:**

Six disease states and 3 stages associated with Coagulopathy in trauma were identified in our study. The hidden Markov model can be useful in identifying latent states by using patients’ time-series multivariate data. The information obtained from the states and stages can be useful in the clinical setting.

**Electronic supplementary material:**

The online version of this article (doi:10.1186/s12911-016-0360-x) contains supplementary material, which is available to authorized users.

## Background

Trauma is the leading cause of death between the ages of 1 to 44 in the United States [1]. Blood loss is the primary cause of these deaths. Understanding how to mitigate this bleeding is essential in saving lives [2]. Following a major traumatic injury involving massive blood loss, patients may become coagulopathic. Coagulopathy is a condition in which blood fails to clot properly, and is associated with a high rate of mortality. Our main objectives in this study are to understand the progression of patient states associated with coagulopathy in trauma, and to identify critical states that might be targeted for interventions.

**Fig. 1:**
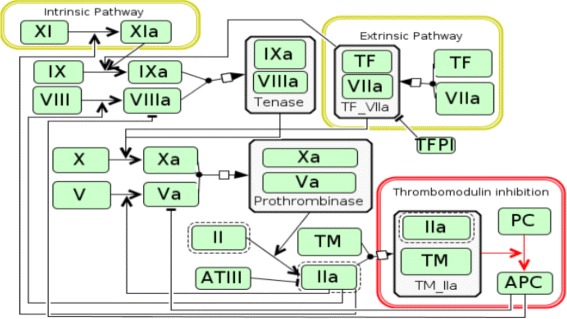
Coagulation cascade. A simplified diagram of the coagulation cascade chemical network, showing both paths of initiation leading to the conversion of prothrombin to thrombin (IIa)

The coagulation cascade [3] is a network of sequential protease activations which ultimately leads to the formation of cross-linked fibrinogen blood clots. The coagulation cascade is shown in Fig. 1, where the roman numerals are blood factors and the roman numerals with an a suffix denotes activated blood factors. The cascade consists of several zymogens, factors II, V, VII, VIII, IX and X. Factor VII is part of the extrinsic pathway [4], which is initiated when factor VII binds to tissue factor (TF) that is exposed during injury. Factor VIII and factor IX are part of the intrinsic pathway, which amplifies the coagulation response. Finally, factor II, factor V and factor X are part of the common pathway, where the two pathways meet. The purpose of the coagulation cascade is to convert prothrombin (Factor II) into thrombin (Factor IIa). Thrombin is the central protein in the coagulation cascade. It is critical for the formation of fibrin links and the activation of platelets, which is essential for forming the plug that covers the injury. If thrombin generation is impaired, it severely affects the body’s ability to heal the wound. In addition to the pro-coagulant proteins in blood plasma, it also contains anti-coagulants such as antithrombin III (ATIII), tissue factor pathway inhibitor (TFPI) and activated protein C (APC) to bring the system back to its original state after the plug has been formed.

In this study, we used time series clinical data for 1413 trauma patients from University of California, San Francisco (UCSF)/San Francisco General Hospital and Trauma Center to identify the disease states. The activity levels of factors II, V, VII, VIII, IX, X, ATIII and protein C, and prothrombin time and partial thromboplastin time were measured at hours 0, 2, 3, 4, 6, 12, 24, 48, 72, 96 and 120 during the course of patients’ hospitalization. We applied the hidden Markov model to the temporal data and distinguished 6 disease states and 3 stages through which patients transition after injury. Specifically, we assumed that the blood factor measurements at each time are generated based on a latent disease state, and that the disease states satisfy the Markov property. With these assumptions we applied the hidden Markov model to infer the hidden states from the patients’ blood data. We examine the properties of each state in detail in the later sections. Furthermore, we discuss their relationships to the blood composition data, and the coagulation cascade, as well as implications derived from our model on clinical practice.

## Methods

### Dataset

Our dataset, from the UCSF/San Francisco General Hospital and Trauma Center, contains 1413 patients admitted to an urban Level I Trauma Center who required intensive care unit (ICU) admission. Blood factors, such as factors II, V, VII, VIII, IX, X, were measured at hours 0, 2, 3, 4, 6, 12, 24, 48, 72, 96 and 120. Other clinical data including prothrombin time and partial thromboplastin time were also recorded at these times. We note that the time course data is very sparse, due to the urgency of the clinical situation.

Due to the interest in analyzing the disease states associated with coagulopathy, we chose 10 blood measurements: Factors II, V, VII, VIII, IX, X, antithrombin III (ATIII), protein C (PC), prothrombin time (PT) and partial thromboplastin time (PTT), to study the progression of states of critical blood protein levels. Factors II, V, VII, VIII, IX, X, antithrombin III and protein C are involved in the coaguloation cascade [3]. The activity levels of these factors have been measured. Prothrombin time and partial thromboplastin time are measurements that are used to characterize the clotting time [5]. Prothrombin time measures the integrity of the extrinsic pathway, and partial thromboplastin time measures the integrity of the intrinsic pathway [4, 6]. Before applying the hidden Markov model, we took the mean of each measurement for each patient within the first 6 hours as hour 0 data, and took the mean of each measurement in hour 12 and hour 24 data as hour 24 data, due to the fact that there are too many missing data from hour 2 to hour 12. Thus, we had data for each patient at 6 possible time points. We removed the patients for whom the data are completely missing in the first 24 hours. After preprocessing, we were left with 1090 patients with a total number of 2176 (patient, time) pairs, where each pair refers to the set of measurements for a single patient at a single time point. The numbers of patients for whom the consecutive (patient, time) pairs were available from hour 0 up to hour 120 are shown in Table 1. After these initial steps, we were left with 25.3 % of data that is missing.

**Table 1:** Number of patients for which consecutive data exists within specified temporal ranges

Temporal range	[0]	[0, 24]	[0, 48]
Number of patients	588	289	42
Temporal range	[0, 72]	[0, 96]	[0, 120]
Number of patients	60	22	89

### Hidden Markov model

Hidden Markov models have been widely used in modeling time series data, especially in speech recognition systems [7], computational molecular biology [8] and other areas of artificial intelligence and pattern recognition [9]. In the following two sections, we briefly introduce the hidden Markov models and their extension to handle missing data.

Supposing for the moment that we do not have missing data in our dataset, we denote the observations at time *t* for a patient as **x**_*t*_, a vector containing the 10 blood data. The hidden Markov model assumes that the observations at time *t* are generated based on a hidden state *z*_*t*_. The state is assumed to be discrete and can take *K* values 1,⋯,*K*. In our scenario, the states are the disease states, which are not directly observed but can be estimated from the data. The hidden Markov model assumes that the hidden states satisfy the Markov property: given the value of *z*_*t*−1_, the probability to be in the current state *z*_*t*_ is independent of all the states prior to *t*−1. Also, given *z*_*t*_, **x**_*t*_ is independent of the states and observations at all other time indices.

**Fig. 2:**
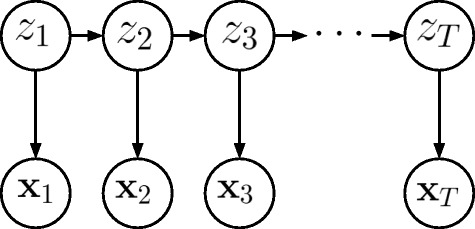
Hidden Markov model. Factorization of the joint probability in a hidden Markov model

Taken together, when there is no missing data, the Markov property implies that for each patient the joint probability distribution of a sequence of states and observations can be factored as:

1 $$\begin{array}{@{}rcl@{}}  p(\mathbf{X}, \mathbf{z}) = p(z_{1})\left[\prod_{t=2}^{T} p(z_{t}|z_{t-1})\right] \left[\prod_{t=1}^{T} p(\mathbf{x}_{t} | z_{t})\right], \end{array} $$

where **X** denotes **x**_1_,⋯,**x**_*T*_ and **z** denotes *z*_1_,⋯,*z*_*T*_. The factorization of the joint probability is shown graphically in Fig. 2. The parameters for the model include an initial state probability *p*(*z*_1_) which sums up to 1, a *K*×*K* transition probability matrix {*P*(*z*_*t*_=*i*|*z*_*t*−1_=*j*)}_*i,j*_, where *i,j*=1,⋯,*K*, and emission probabilities *p*(**x**_*t*_|*z*_*t*_). For each of the *K* state, there is a set of emission probabilities that governs the observed data **x**_*t*_ given the hidden state *z*_*t*_. We assume the emission probability for each state to be a multivariate Gaussian distribution. Thus for each state, we have a mean vector and a covariance matrix as model parameters. The hidden Markov model assumes that these parameters are not dependent on *t*. In order to estimate the parameters, we need to maximize the likelihood, which can be obtained by marginalizing out **z** in (1) as

2 $$\begin{array}{@{}rcl@{}}  p(\mathbf{X}) = \sum_{\mathbf{z}}p(\mathbf{X}, \mathbf{z}). \end{array} $$

However, this likelihood function is very difficult to optimize directly.

Expectation maximization (EM) algorithm, also called the Baum–Welch algorithm [10] in the setting of the hidden Markov model, is a standard method to infer the parameters in the hidden Markov model [11]. The EM algorithm is an efficient framework to maximize the likelihood function with latent variables or missing data. The idea of the EM algorithm is to maximize the expectation of the complete-data log likelihood function with respect to the posterior distribution of the latent data through an iterative process. It starts with some initial values for the parameters. In the E step, it evaluates the expectation of the complete-data log likelihood with respect to the posterior distribution of *z* using the current parameter. Then in the M step, it maximizes the expectation of the complete-data log likelihood function evaluated in the E step with respect to the parameters. The EM algorithm iteratively computes the E and M steps until convergence.

We note that the hidden Markov model does not assume that the observed measurements follow the Markov property. Actually, the current measurements are dependent on all the previous measurements, even with the assumption that the hidden states follow the Markov property. Also, note that due to the non-convexity of the problem, the EM algorithm can only find a local minimum rather than a global minimum. In most cases, a local minimum is sufficient in practice [10].

### Hidden Markov model with missing data

Before proceeding to discuss missing data, we must distinguish two types of missingness. The first type refers to an incomplete set of measurements at a single time point *t*_0_ (it may still contain other measurements at *t*_0_ or at later time points). The other type of missing data refers to data missing for a patient completely after some time point *T*_*s*_. In our data, the first type of missing data occurs mainly because of the urgency of the clinical situation, and can be regarded as missing at random (MAR) [12]. This type of missing data requires special care in the hidden Markov model. The second type of missing data is mainly due to censoring, for example where patients die, get discharged, or recover completely and fall out of the study. Data with this type of missingness is not typically considered as missing data, and the hidden Markov model can be applied to it directly. We did not count the second type missingness when calculating the percentage of missing data.

In the case of MAR data, both latent variables **z** and missing data **X**^*mis*^ are unobserved. EM algorithm can still be used, however both the E and M steps must be modified. In the E step, the algorithm evaluates the expectation of the complete-data log likelihood function with respect to the posterior distribution of both **z** and **X**^*mis*^. The expectation of the complete-data log likelihood function can be separated into two parts: the expectations with respect to the latent states, which is the same as the E step when missing data are ignored, and the expectations with respect to the missing data. Because we assume **X** have multivariate Gaussian distributions, the posterior distributions of the missing data are conditional distributions of Gaussian distribution given the observed data, which also have Gaussian distributions. Thus the mean vectors and covariance matrices of the posterior distribution can be calculated. They can be easily obtained by using the sweep operator and sweeping the augmented covariance matrix [12]. In the M step, the algorithm updates the parameters by maximizing the evaluated expectations of the complete-data log likelihood function. Detailed derivations are provided in the Additional file 1.

Once we have obtained the parameters, we can infer the most probable sequence of hidden states (the trajectory of states) for each patient, by maximizing the posterior probability of *p*(**z**|**X**), via the Viterbi algorithm [13]. With the parameters, the model can also be used to estimate states and missing data in a clinical setting where the blood factor measurements are not typically available. For example, with the temporal measurements of PT and PTT, we can use the learnt model to infer the disease states and estimate the values of the blood factors. We can also make predictions of the patients’ future states and trajectories.

### Model customization

**Fig. 3:**
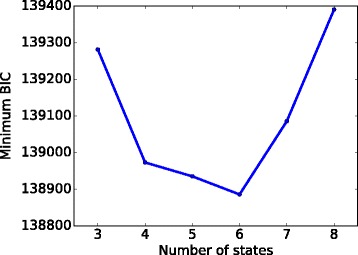
Choosing the number of states. For the number of states from 3 to 8, we ran the model corresponding to each number of states 50 times, and plotted the minimum BIC for each number of states. We chose the model with 6 states because it can achieve the lowest BIC

The hidden Markov model requires a pre-specified number of states. To identify the best model, we looped through the possible numbers of states from 3 to 8, found the hidden Markov model for each candidate number of states 50 times with different seeds, and calculated the BIC (Bayesian Information Criterion) [14] for each run. The BIC is calculated as

3 $$\begin{array}{@{}rcl@{}} \textup{BIC} = -2 \cdot \textup{log}p(\mathbf{X}) + \textup{n\_params} \cdot \textup{log}(\textup{n\_data}), \end{array} $$

where n_params is the number of parameters. n_params is *K*−1 (for the initial probability) plus *K*×(*K*−1) (for the transition probability matrix) plus *d*×*K* and $\frac {d \times (d+1)}{2} \times K$ (for the mean and covariance matrix for the Gaussian emission probability), where *d* is the number of features, which is 10 in our case. The minimums of the BICs for each number of states are shown in Fig. 3. We chose the model with the minimum BIC as our best model. That model has 6 states.

## Results

### Model results

**Fig. 4:**
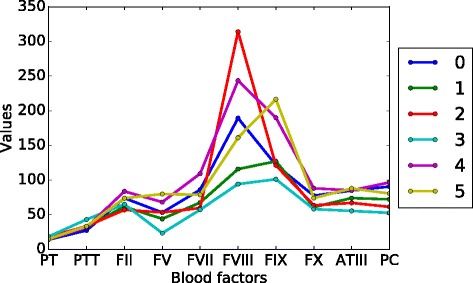
Mean values of blood factors in each state

**Fig. 5:**
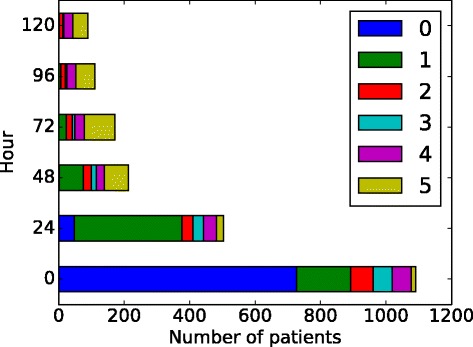
Number of patients in each state across time

After deciding on the best model, we found the 6 states, State 0, State 1,..., State 5, and the parameters in the model. The initial probability is shown in Table 2 and the transition matrix is shown in Table 3, where State *n* is referred to S*n*. The means of the emission probabilities for each state are shown in Table 4 and Fig. 4. We summed up *P*(*z*_*t*_|**x**_*t*_) across all patients at each time *t*, which gives us the total number of patients at each state at *t*, shown in Fig. 5. We have also obtained the trajectories for each patient. We used the trajectories to calculate the probabilities from each state to death or discharge. We attributed mortality or discharge to a state if it was the last state prior to death/discharge within a 5-day window. A 5-day window, instead of a 24 h one, is used because very few patients died or got discharged within 24 h after their last measurements. Also, in order to reduce the bias caused by censoring, we only used the trajectories that have more than one temporal measurement to calculate this probability (Table 5). Of these trajectories, the most common multi-state trajectories are (State 0, State 1, discharge) and (State 0, State 1, State 5, discharge).

**Table 2:** The initial probabilities of the hidden Markov model

States	S0	S1	S2	S3	S4	S5
Probability	0.666	0.151	0.063	0.054	0.054	0.012

**Table 3:** State transition matrix. These are the probabilities of moving from one state to another, in the next 24 h time window

	S0	S1	S2	S3	S4	S5
S0	0.156	0.768	0.017	0.013	0.046	0
S1	0	0.57	0.05	0.004	0.017	0.358
S2	0	0.121	0.757	0	0.07	0.052
S3	0	0.095	0.037	0.772	0.047	0.049
S4	0	0	0	0	1	0
S5	0	0	0	0	0.056	0.944

**Table 4:** The mean of the emission probabilities

	S0	S1	S2	S3	S4	S5
PT	13.883	15.648	17.67	18.31	14.287	14.712
PPT	27.195	31.9	33.376	42.996	31.609	33.251
FII	73.876	61.495	56.856	65.047	83.602	73.627
FV	53.025	43.897	53.199	23.272	68.063	79.825
FVII	85.94	67.406	59.516	57.084	109.144	78.721
FVIII	189.304	115.786	314.08	94.138	243.429	160.961
FIX	121.607	126.985	121.193	101.069	189.968	216.546
FX	77.386	60.911	63.479	58.019	88.028	73.885
ATIII	84.848	73.927	66.948	55.363	85.048	87.858
PC	90.467	72.025	61.216	52.578	96.591	80.464

**Table 5:** The probabilities from each state directly to death and discharge within 5 days

States	S0	S1	S2	S3	S4	S5
*P*(*death*)	0.167	0.316	0.375	0.6	0.286	0.333
*P*(*discharge*)	0.833	0.684	0.625	0.4	0.714	0.667

### Blood progression states

**Fig. 6:**
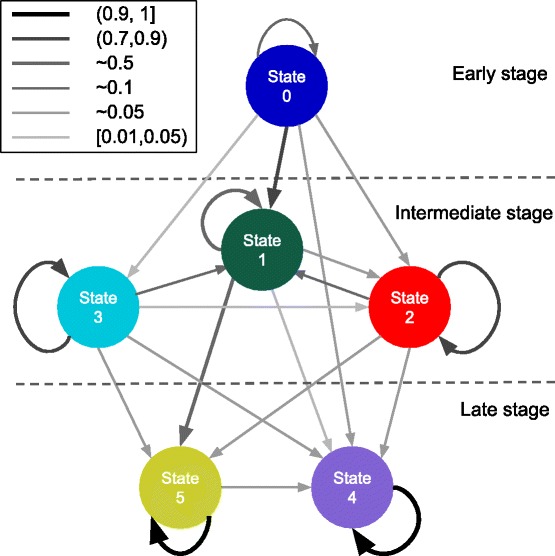
Transition matrix diagram. Transition probabilities between states. A wider and more intense *color arrow* indicates higher transition probability

From Fig. 5 and Table 3, we found that the states fall into 3 stages: early stage (State 0), intermediate stage (State 1, 2 and 3) and late stage (State 4 and 5). We note that the temporal characterization of the stages describes the temporal relationship between the stages, but not the absolute time of occurrence of any of the states. For example, if a state in the intermediate stage is going to change to a different state, it will move into another state in the intermediate stage or a state in the late stage, but it will not move into a state in the early stage. However, that is not to say that a patient cannot arrive already in a state from the intermediate stage or the late stage as that does occur in our data. The state diagram and stage separation are shown in Fig. 6, where a wider and more intense color arrow indicates higher transition probability.

Apart from temporal relationships, each state can be characterized by its blood profiles (Fig. 4), its relationships with the other states (Table 3) and its mortality rate (Table 5). We let *P*_*m*→*n*_ denotes *P*(*z*_*t*_=*n*|*z*_*t*−1_=*m*). State 0, the only state in the early stage, is characterized by having the fastest clot times and average blood factor levels. We refer to the state in which patients are more likely to remain for one time interval as a *stable* state; otherwise as an *unstable* state. State 0 is the most unstable state, in that *P*_0→0_=0.16. Patients in this state are most likely to transition to states in the intermediate stage, with the highest probability of going to State 1 (*P*_0→1_=0.77). Since this state is the most unstable, it is the most critical state to influence the patient’s trajectory and probably the most promising for intervention.

There are 3 states in the intermediate stage (States 1, 2 and 3). The states in this stage are more stable than the early stage state, but less stable than the late stage states. The states in this stage have the largest variation of mortality rates and therefore are the best indicators in terms of patient outcome. State 0 transitions to State 1 at the highest probability. It is the state with the lowest rate of mortality (*P*_1→*death*_=0.316) in the intermediate stage. State 1 is characterized by average clot times and slightly below-average factor levels. This is probably due to the initial coagulation process using up some of the blood factors, which slows down the clot times. It is a relatively unstable state, with a relatively low probability of remaining in the same state (*P*_1→1_=0.57). If the patient moves to a different state, he/she is most likely to move to State 5 (*P*_1→5_=0.36) or to one of the other intermediate stages at a much lower probability (*P*_1→2_=0.05, *P*_1→3_=0.004). The next lowest mortality rate among the intermediate stage states is State 2 (*P*_2→*death*_=0.375). It is characterized by high clot times and low blood factors, with the exception of factor VIII which is high. State 2 is a relatively stable state (*P*_2→2_=0.76) but is most likely to transition to State 1 (*P*_2→1_=0.12) and less likely to transition to the late stage states. State 3 is the state with the highest mortality rate (*P*_3→*death*_=0.6) and is characterized by having the longest clot times and the lowest blood factor levels. It is also a relatively stable state that is most likely to transition to State 1 but can also transition to the states in the late stage.

Lastly, there are two states in the late stage (States 4, 5). Both of these states are very stable and have low mortality. Both of these states have fast clot times and high factor levels. The differences between these states primarily arise from the blood factor levels, in which State 4 has higher levels of prothrombin, Factors VII, VIII, X and protein C, while State 5 has higher levels of factors V and IX and ATIII. Patients in State 4 are probably healthier as they have lower rate of mortality, shorter clot times and are more stable (State 5 can transition to State 4, but not vice versa).

It is important to note that the probabilities of the transition matrix do not include the transition to death or discharge, due to the requirement of equally spaced time points in the hidden Markov model. Furthermore, patients that recover quickly are less likely to get the blood sample showing the healthy results. It is most likely for this reason that the probability to transition to the healthiest late state (State 4) is higher for the sicker intermediate states (2 and 3) than the healthier one (State 1). It should also be mentioned that the mortality rates include causes not associated with coagulation impairment. This, combined with the fact that healthier patients are less likely to have blood factor data, are reasons why the “healthier” late stage states can have a higher mortality probability than State 0.

## Discussion

### Relationship to the coagulation process

The coagulation potential of a trauma patient can undergo several trajectories as shown by the separation of the 6 states of our Hidden Markov Model into 3 stages. In the early stages, the coagulation factors are still plentiful and are more likely to have activated factors and their associated enzyme complexes such as prothrombinase and tenase in their plasma, causing their clot times to be the lowest among all of the states. From the early stage state 0, the patient will likely transition into another state depending on the severity of their injury, the patient’s overall health and the effectiveness of medical interventions. This state has the highest potential for intervention, as it is the least stable. The states in the intermediate stages are characterized by slower clot times and lower factor levels. This is likely to come from blood loss and factor consumption from the coagulation process. These states represent the impairment to the coagulation process caused by the injury. Because of this, the states of the intermediate stage are the most predictive of patient outcome, as patients in states 1 and 2 have much better outcomes than patients in state 3. The relatively high stability of states 2 and 3 may be an indication of how resilient these states are to intervention. The late stage states have fast clot times and high factor levels, which are indications that the patient’s coagulation potential has been restored.

Another observation can be made from the transition matrix about the trajectory of the coagulation potential of a trauma patient. The state that the patient moves to after leaving the early stage will usually be the most severe state that the patient will be in, in terms of impairment of blood coagulation. After moving from the early stage to the intermediate stage, there are typically no transitions from a healthier state to a sicker state with the small exception of the small probability that State 1 transitions to State 2 (*P*_1→2_=0.05), where State 1 has a lower rate of mortality than State 2. This observation may suggest some interesting clinical assumptions.

### Clinical consequences

The Hidden Markov Model of the impact of trauma on coagulation provides an improved understanding that can shape the way we treat trauma patients. If blood data are measured for patients in an ICU, our learnt model can be used to quickly assess the current state of the patient and predict their future states and trajectories. Although the blood factor data is not usually available, microfluid technology [15] is improving at a very rapid pace, that it will be possible to easily obtain this data in the foreseeable future. When only some of the measurements are available, our learnt model can still estimate the patient’s state and the measurements of other blood data. Based on the critical states we have found, patients can be treated with the most suitable interventions to decrease their risks of death. The knowledge generated from the model can be particularly valuable for the evaluation and design of healthcare provider protocols, and the selections to allocate resources to obtain the best outcomes. Furthermore, the methods in this paper provide a framework that can be extended to model other diseases with complex patient trajectories.

## Conclusions

In summary, we applied a hidden Markov model to the blood measurements for 1090 patients and identified 6 disease states and 3 stages. We showed the properties of each state, their transition probabilities, and discussed the clinical relevance of the states.

## Additional file

Additional file 1 Identification of Disease States Associated with Coagulopathy in Trauma. (PDF 196 kb)

## Abbreviations

ICU: Intensive care unit
UCSF: University of California, San Francisco
IRB: Institutional Review Board
ATIII: Antithrombin III
TFPI: Tissue factor pathway inhibitor
APC: Activated protein C
PC: Protein C
PT: Prothrombin time
PTT: Partial thromboplastin time
EM: Expectation maximization
MAR: Missing at random
BIC: Bayesian information criterion
