# Supplementary material for: Identification of disease states associated with coagulopathy in trauma
Source: BMC Med Inform Decis Mak. 2016 Sep 22;16:124. doi: 10.1186/s12911-016-0360-x (PMC5034569; doi:10.1186/s12911-016-0360-x)
Supplement: Additional file 1 — Identification of Disease States Associated with Coagulopathy in Trauma. (PDF 196 kb) [file 12911_2016_360_MOESM1_ESM.pdf]

Supplementary Material for "Identification of Disease States Associated with Coagulopathy in Trauma"

Yuanyang Zhang[1], Tie Bo Wu[2], Bernie J Daigle Jr[3], Mitchell Cohen[4] and Linda Petzold[1]
[1]*Department of Computer Science, University of California, Santa Barbara*
[2]*Department of Mechanical Engineering, University of California, Santa Barbara*
[3]*Department of Biological Sciences and Computer Science, University of Memphis*
[4]*Department of Surgery, University of California, San Francisco*

EM ALGORITHM FOR HIDDEN MARKOV MODEL

Without missing data

We begin with the situation where there is no missing data in the dataset. Once we have derived the EM algorithm, the situation where there are missing data can be handled with few modifications.

Suppose that we have temporal measurements $\mathbf{x}_1, \mathbf{x}_2, \cdots, \mathbf{x}_T$, and we assume that their latent states are $\mathbf{z}_1, \mathbf{z}_2, \cdots, \mathbf{z}_T$. Suppose that there are K states in the hidden Markov model. Each $\mathbf{x}_t$ is a D-dimensional vector of observed data, and $\mathbf{z}_t$ is a K-dimensional binary vector with components that add up to 1, where $z_{tj} = 1$ means $\mathbf{x}_t$ is at state j. The joint probability distribution for hidden Markov model is given by

$$p(\mathbf{x}_1, \cdots, \mathbf{x}_T, \mathbf{z}_1, \cdots, \mathbf{z}_T) = p(\mathbf{z}_1) \left[\prod_{t=2}^{T} p(\mathbf{z}_t | \mathbf{z}_{t-1}) \right] \prod_{t=1}^{T} p(\mathbf{x}_t | \mathbf{z}_t) \tag{1}$$

We represent the initial probability by a vector of probabilities $\boldsymbol{\pi}$ with elements $\pi_k \equiv p(z_{1k} = 1)$, and $\sum_k^K \pi_k = 1$. We represent the transition probability matrix as $\mathbf{A}$, where $A_{jk} \equiv p(z_{tk} = 1 | z_{t-1,j} = 1)$, and $\sum_k^K A_{jk} = 1$. Thus we have

$$p(\mathbf{z}_1 | \boldsymbol{\pi}) = \prod_{k=1}^{K} \pi_k^{z_{1k}}, \tag{2}$$

and

$$p(\mathbf{z}_t | \mathbf{z}_{t-1}, \mathbf{A}) = \prod_{k=1}^{K} \prod_{j=1}^{K} A_{jk}^{z_{t-1,j} z_{tk}}. \tag{3}$$

We assume the emission probability $p(\mathbf{x}_t | \mathbf{z}_t)$ follows a Gaussian distribution. We have $\mathbf{x}_t | (z_{tk} = 1) \sim \mathcal{N}(\boldsymbol{\mu}_k, \boldsymbol{\Sigma}_k)$, where $\boldsymbol{\mu}_k$ and $\boldsymbol{\Sigma}_k$ are the mean vector and covariance matrix for state k, respectively. Thus we have

$$p(\mathbf{x}_t | \mathbf{z}_t, \boldsymbol{\mu}, \boldsymbol{\Sigma}) = \prod_{k=1}^{K} p(\mathbf{x}_t | \boldsymbol{\mu}_k, \boldsymbol{\Sigma}_k)^{z_{tk}}. \tag{4}$$

We can write down the log of the joint probability distribution given the parameters $\boldsymbol{\theta} = (\boldsymbol{\pi}, \mathbf{A}, \boldsymbol{\mu}, \boldsymbol{\Sigma})$ as

$$\log p(\mathbf{x}_1, \cdots, \mathbf{x}_T, \mathbf{z}_1, \cdots, \mathbf{z}_T) = \sum_{k=1}^{K} z_{1k} \log \pi_k + \sum_{t=2}^{T} \sum_{j=1}^{K} \sum_{k=1}^{K} z_{t-1,j} z_{tk} \log A_{jk} + \sum_{t=1}^{T} \sum_{k=1}^{K} z_{tk} \log p(\mathbf{x}_t | \boldsymbol{\mu}_k, \boldsymbol{\Sigma}_k). \tag{5}$$

The EM algorithm [1] is typically used to estimate the parameters in the hidden Markov model. The EM algorithm tries to maximize the expectation of the complete-data log likelihood function (5) given the posterior distribution of $\mathbf{Z}$, which is

$$\mathcal{Q}(\boldsymbol{\theta}, \boldsymbol{\theta}^{old}) = \sum_{k=1}^{K} \mathbf{E}_{\mathbf{z}}[z_{1k}] \log \pi_k + \sum_{t=2}^{T} \sum_{j=1}^{K} \sum_{k=1}^{K} \mathbf{E}_{\mathbf{z}}[z_{t-1,j} z_{tk}] \log A_{jk} + \sum_{t=1}^{T} \sum_{k=1}^{K} \mathbf{E}_{\mathbf{z}}[z_{tk}] \log p(\mathbf{x}_t | \boldsymbol{\mu}_k, \boldsymbol{\Sigma}_k). \tag{6}$$

In the E step, we use the current parameters to evaluate the expectation of the complete-data log likelihood function, and in the M step, we maximize the expectation of the complete-data log likelihood function and update the parameters. We need to evaluate the expectations $\mathbf{E_z}[\mathbf{z}_t] = p(\mathbf{z}_t|\mathbf{X}, \boldsymbol{\theta})$ and $\mathbf{E_z}[\mathbf{z}_{t-1}\mathbf{z}_t] = p(\mathbf{z}_{t-1}, \mathbf{z}_t|\mathbf{X}, \boldsymbol{\theta})$. We write $\gamma(z_{tk}) = \mathbf{E_Z}[z_{tk}]$ and $\xi(z_{t-1,j}, z_{tk}) = \mathbf{E_z}[z_{t-1,j}z_{tk}]$. The forward-backward algorithm [1] is used to estimate them. The forward-backward algorithm basically calculates

$$\begin{aligned} \alpha(\mathbf{z}_t) &\equiv p(\mathbf{x}_1, \cdots, \mathbf{x}_t, \mathbf{z}_t) && (7)\\ \beta(\mathbf{z}_t) &\equiv p(\mathbf{x}_{t+1}, \cdots, \mathbf{x}_T|\mathbf{z}_t). && (8) \end{aligned}$$

Following the forward algorithm, we have

$$\alpha(\mathbf{z}_t) = p(\mathbf{x}_t|\mathbf{z}_t) \sum_{\mathbf{z}_{t-1}} \alpha(\mathbf{z}_{t-1}) p(\mathbf{z}_t|\mathbf{z}_{t-1}), \tag{9}$$

where the initial condition is given by

$$\alpha(\mathbf{z}_1) = p(\mathbf{x}_1, \mathbf{z}_1) = \prod_k^K \left\{\pi_k p(\mathbf{x}_1|\boldsymbol{\mu}_k, \boldsymbol{\Sigma}_k)\right\}. \tag{10}$$

Following the backward algorithm, we have

$$\beta(\mathbf{z}_t) = \sum_{\mathbf{z}_{t+1}} \beta(\mathbf{z}_{t+1}) p(\mathbf{x}_{t+1}|\mathbf{z}_{t+1}) p(\mathbf{z}_{t+1}|\mathbf{z}_t), \tag{11}$$

where the initial condition is $\beta(\mathbf{z}_T) = \mathbf{1}$. And we have

$$\gamma(\mathbf{z}_t) = \frac{\alpha(\mathbf{z}_t)\beta(\mathbf{z}_t)}{p(\mathbf{X})}, \tag{12}$$

where $p(\mathbf{X})$ is the likelihood function and can be obtained by

$$p(\mathbf{X}) = \sum_{\mathbf{z}_T} \alpha(\mathbf{z}_T), \tag{13}$$

and

$$\xi(\mathbf{z}_{t-1}, \mathbf{z}_t) = \frac{\alpha(\mathbf{z}_{t-1}) p(\mathbf{x}_t|\mathbf{z}_t) p(\mathbf{z}_t|\mathbf{z}_{t-1}) \beta(\mathbf{z}_t)}{p(\mathbf{X})}. \tag{14}$$

We have omitted all the derivations of the forward-backward algorithm. For detailed derivations, please refer to [1].

Once we have obtained the $\gamma(\mathbf{z}_t)$ and $\xi(\mathbf{z}_{t-1}, \mathbf{z}_t)$, we can go to the M step, where we use these expectations to estimate the parameters. It follows the same solution steps for maximizing the likelihood of multinomial distribution and Gaussian distribution. We have

$$\begin{aligned} \pi_k &= \frac{\gamma(z_{1k})}{\sum_{j=1}^K \gamma(z_{1k})}, && (15)\\ A_{jk} &= \frac{\sum_{t=2}^T \xi(z_{t-1,j} z_{tk})}{\sum_{l=1}^K \sum_{t=2}^T \xi(z_{t-1,j} z_{tl})}, && (16)\\ \boldsymbol{\mu}_k &= \frac{\sum_{t=1}^T \gamma(z_{tk}) \mathbf{x}_t}{\sum_{t=1}^T \gamma(z_{tk})}, && (17)\\ \boldsymbol{\Sigma}_k &= \frac{\sum_{t=1}^T \gamma(z_{tk}) (\mathbf{x}_t - \boldsymbol{\mu}_k)(\mathbf{x}_t - \boldsymbol{\mu}_k)^{\mathrm{T}}}{\sum_{t=1}^T \gamma(z_{tk})}. && (18) \end{aligned}$$

So the EM algorithm works as follows:

1. Initialize the parameters $\boldsymbol{\theta}$. We can use K-means algorithm and the resulting K clusters to initialize $\boldsymbol{\mu}$'s and $\boldsymbol{\Sigma}$'s. We also initialize the log-likelihood to be $-\infty$.

2. **E step** Use the parameters $\boldsymbol{\theta}^{old}$ to calculate $\gamma(\mathbf{z}_t)$ and $\xi(\mathbf{z}_{t-1}, \mathbf{z}_t)$ following the forward-backward algorithm. We also calculate the new log-likelihood.

3. **M step** Update the parameters $\boldsymbol{\theta}$ using $\gamma(\mathbf{z}_t)$ and $\xi(\mathbf{z}_{t-1}, \mathbf{z}_t)$.

4. If the absolute difference between the old and new log-likelihood is below a certain threshold, terminate the EM algorithm and output $\boldsymbol{\theta}$. Update the old log-likelihood otherwise.

Notice that we have illustrated how to estimate $\boldsymbol{\theta}$ from only one sequence of temporal measurements, for the purpose of clarity. When there are multiple sequences of temporal measurements, we need to evaluate the γ and ξ for each sequence in the E step. In the M step, we average over all the sequences to obtain the new $\boldsymbol{\theta}$. We will use the same way to demonstrate the EM algorithm for hidden Markov model with MAR data.

With missing at random (MAR) data

When there are MAR data [2] in the dataset, each $\mathbf{x}_t$ can be written as $(\mathbf{x}_t^{obs}, \mathbf{x}_t^{mis})$, where $\mathbf{x}_t^{obs}$ and $\mathbf{x}_t^{mis}$ are the observed portion and the missing portion of of $\mathbf{x}_t$, respectively. So the corresponding log of the joint probability distribution is given by

$$\begin{aligned}&\log p(\mathbf{x}_1^{obs}, \cdots, \mathbf{x}_T^{obs}, \mathbf{x}_1^{mis}, \cdots, \mathbf{x}_T^{mis}, \mathbf{z}_1, \cdots, \mathbf{z}_T) \\ =& \sum_{k=1}^{K} z_{1k}\log\pi_k + \sum_{t=2}^{T}\sum_{j=1}^{K}\sum_{k=1}^{K} z_{t-1,j}z_{tk}\log A_{jk} + \sum_{t=1}^{T}\sum_{k=1}^{K} z_{tk}\log p(\mathbf{x}_t|\boldsymbol{\mu}_k, \boldsymbol{\Sigma}_k).\end{aligned} \tag{19}$$

The corresponding expectation of the complete-data log likelihood function given the expectation of the posterior distribution of $\mathbf{Z}$ and $\mathbf{X}^{mis}$ is given by

$$\mathcal{Q}(\boldsymbol{\theta}, \boldsymbol{\theta}^{old}) = \sum_{k=1}^{K} \mathbf{E}_{\mathbf{z},\mathbf{x}^{mis}}[z_{1k}]\log\pi_k + \sum_{t=2}^{T}\sum_{j=1}^{K}\sum_{k=1}^{K} \mathbf{E}_{\mathbf{z},\mathbf{x}^{mis}}[z_{t-1,j}z_{tk}]\log A_{jk} + \sum_{t=1}^{T}\sum_{k=1}^{K} \mathbf{E}_{\mathbf{z},\mathbf{x}^{mis}}[z_{tk}\log p(\mathbf{x}_t|\boldsymbol{\mu}_k, \boldsymbol{\Sigma}_k)]. \tag{20}$$

Because the expectations of $z_{tk}, z_{t-1,j}z_{tk}$ only depend on $\mathbf{Z}$ and the expectations of $\log p(\mathbf{x}_t|\boldsymbol{\mu}_k, \boldsymbol{\Sigma}_k)$ only depend on $\mathbf{X}^{mis}$, we have

$$\mathcal{Q}(\boldsymbol{\theta}, \boldsymbol{\theta}^{old}) = \sum_{k=1}^{K} \mathbf{E}_{\mathbf{z}}[z_{1k}]\log\pi_k + \sum_{t=2}^{T}\sum_{j=1}^{K}\sum_{k=1}^{K} \mathbf{E}_{\mathbf{z}}[z_{t-1,j}z_{tk}]\log A_{jk} + \sum_{t=1}^{T}\sum_{k=1}^{K} \mathbf{E}_{\mathbf{z}}[z_{tk}]\mathbf{E}_{\mathbf{x}^{mis}}[\log p(\mathbf{x}_t|\boldsymbol{\mu}_k, \boldsymbol{\Sigma}_k)]. \tag{21}$$

Note that we have already derived $\mathbf{E}_{\mathbf{z}}[\mathbf{z}_t]$ and $\mathbf{E}_{\mathbf{z}}[\mathbf{z}_{t-1}\mathbf{z}_t]$, which are $\gamma(\mathbf{z}_t)$ and $\xi(\mathbf{z}_{t-1}, \mathbf{z}_t)$ in (12) and (14). The only difference is that the $p(\mathbf{x}_t|\mathbf{z}_t)$ in equations (9) to (14) should be replaced with $p(\mathbf{x}_t^{obs}|\mathbf{z}_t)$. Because the marginal distribution over a subset of multivariate Gaussian is still Gaussian distribution, we have $\mathbf{x}_t^{obs}|(z_{tk} = 1) \sim \mathcal{N}(\boldsymbol{\mu}_k^{obs}, \boldsymbol{\Sigma}_k^{obs})$, where $\boldsymbol{\mu}_k^{obs}$ and $\boldsymbol{\Sigma}_k^{obs}$ are the mean vector and covariance matrix after dropping the missing variables from $\boldsymbol{\mu}_k$ and $\boldsymbol{\Sigma}_k$. So $p(\mathbf{x}_t^{obs}|\boldsymbol{\mu}_k, \boldsymbol{\Sigma}_k) = p(\mathbf{x}_t^{obs}|\boldsymbol{\mu}_k^{obs}, \boldsymbol{\Sigma}_k^{obs})$ and therefore we can obtain $p(\mathbf{x}_t^{obs}|\mathbf{z}_t)$. If all the data are missing at $\mathbf{x}_t$, $p(\mathbf{x}_t^{obs}|\boldsymbol{\mu}_k, \boldsymbol{\Sigma}_k)$ is simply 1.

In order to write down the E step, we need to obtain the $\mathbf{E}_{\mathbf{x}^{mis}}[\log p(\mathbf{x}_t|\boldsymbol{\mu}_k, \boldsymbol{\Sigma}_k)], k = 1, \cdots, K$. Note that the posterior distribution of $\mathbf{x}_t^{mis}$ is a conditional distribution of a subset of variables from a multivariate Gaussian distribution, which is also a Gaussian distribution. Because the complete $\mathbf{X}$ belong to the regular exponential family, we only need to evaluate the expectation of the sufficient statistics, which are $\sum_{t=1}^{T} x_{tj}$, where $j = 1, \cdots, D$, and $\sum_{t=1}^{T} x_{tj}x_{tl}$, where $j, l = 1, \cdots, D$. Based on [2], the E step for kth state includes

$$\mathbf{E}_{\mathbf{x}^{mis},k}\left[\sum_{t=1}^{T} x_{tj}\right] = \sum_{t=1}^{T} \widehat{x}_{tj}^{(k)}, \quad j = 1, \cdots, D \tag{22}$$

and

$$\mathbf{E}_{\mathbf{x}^{mis},k}\left[\sum_{t=1}^{T} x_{tj}x_{tl}\right] = \sum_{t=1}^{T}(\widehat{x}_{tj}^{(k)}\widehat{x}_{tl}^{(k)} + \widehat{c}_{jln}^{(k)}), \quad j, l = 1, \cdots, D \tag{23}$$

where

$$\widehat{x}_{tj}^{(k)} = \begin{cases} x_{tj}, & \text{if } x_{tj} \text{ is observed.} \\ \mathbf{E}(x_{tj}|\mathbf{x}_t^{obs}, \boldsymbol{\mu}_k, \boldsymbol{\Sigma}_k), & \text{otherwise.} \end{cases} \tag{24}$$

and

$$\widehat{c}_{jln}^{(k)} = \begin{cases} 0, & \text{if } x_{tj} \text{ or } x_{tl} \text{ is observed.} \\ Cov(x_{tj}, x_{tl}|\mathbf{x}_t^{obs}, \boldsymbol{\mu}_k, \boldsymbol{\Sigma}_k), & \text{otherwise,} \end{cases} \tag{25}$$

where $\mathbf{E}(x_{tj}|\mathbf{x}_t^{obs}, \boldsymbol{\mu}_k, \boldsymbol{\Sigma}_k)$ and $Cov(x_{tj}, x_{tl}|\mathbf{x}_t^{obs}, \boldsymbol{\mu}_k, \boldsymbol{\Sigma}_k)$ are the conditional mean vector and covariance matrix of the missing data $\mathbf{x}_t^{mis}$ given the observed data $\mathbf{x}_t^{obs}$ and the current parameters $\boldsymbol{\mu}_k, \boldsymbol{\Sigma}_k$.

To evaluate $\mathbf{E}(x_{tj}|\mathbf{x}_t^{obs}, \boldsymbol{\mu}_k, \boldsymbol{\Sigma}_k)$ and $Cov(x_{nj}, x_{nl}|\mathbf{x}_t^{obs}, \boldsymbol{\mu}_k, \boldsymbol{\Sigma}_k)$, we need to obtain the conditional distribution of the missing features given the set of the observed features. The $\widehat{\mathbf{x}}_t^{(k)}$ is basically an imputed $\mathbf{x}_t$ in state k where the missing data are replaced by the conditional mean. An easy way to compute the conditional distribution is to use the sweep operator to sweep the augmented covariance matrix [2]. In practice, we could calculate all the missing patterns in the dataset in advance, and in each E step, for each state, we calculate the swept matrices for all missing patterns. Then for each $\mathbf{x}_t$, we can estimate its conditional mean vector and covariance matrix by using the corresponding swept matrix.

In the M step, we update the parameters by using the expectation we evaluated in the E step. We can do the same with equations (15) and (16) to update of π_k and A_{jk}. To update $\boldsymbol{\mu}_k$ and $\boldsymbol{\Sigma}_k$, we have

$$\boldsymbol{\mu}_k = \frac{\sum_{t=1}^{T} \gamma(z_{tk}) \widehat{\mathbf{x}}_t^{(k)}}{\sum_{t=1}^{T} \gamma(z_{tk})}, \tag{26}$$

$$\begin{aligned} \boldsymbol{\Sigma}_k &= \frac{\sum_{t=1}^{T} \gamma(z_{tk}) \left\{ \mathbf{E}\left[(\widehat{\mathbf{x}}_t^{(k)})(\widehat{\mathbf{x}}_t^{(k)})^{\mathrm{T}} \right] - \boldsymbol{\mu}_k \boldsymbol{\mu}_k{}^{\mathrm{T}} \right\}}{\sum_{t=1}^{T} \gamma(z_{tk})} \\ &= \frac{\sum_{t=1}^{T} \gamma(z_{tk}) \left[(\widehat{\mathbf{x}}_t^{(k)} - \boldsymbol{\mu}_k)(\widehat{\mathbf{x}}_t^{(k)} - \boldsymbol{\mu}_k)^{\mathrm{T}} + \widehat{C}_t^{(k)} \right]}{\sum_{t=1}^{T} \gamma(z_{tk})}, \end{aligned} \tag{27}$$

where

$$\widehat{C}_t^{(k)} = \left[\widehat{c}_{jlt}^{(k)} \right]_{j,l=1,\cdots,D}. \tag{28}$$

So the EM algorithm works as follows:

1. Initialize the parameters $\boldsymbol{\theta}$. We can use K-means and the resulting K clusters to initialize $\boldsymbol{\mu}$'s and $\boldsymbol{\Sigma}$'s. Note that taking means of missing data may cause trouble, we can use all complete $\mathbf{x}$'s in each cluster to initialize $\boldsymbol{\mu}$'s and $\boldsymbol{\Sigma}$'s. We also initialize the log-likelihood to be $-\infty$.

2. **E step** Use the parameters $\boldsymbol{\theta}^{old}$ to calculate $\gamma(\mathbf{z}_t)$ and $\xi(\mathbf{z}_{t-1}, \mathbf{z}_t)$ and the new log-likelihood following the forward-backward algorithm. We also calculate $\widehat{\mathbf{x}}_t^{(k)}$ and $\widehat{C}_t^{(k)}$ from equations (24) and (25).

3. **M step** Update the parameters $\boldsymbol{\theta}$ using $\gamma(\mathbf{z}_t)$, $\xi(\mathbf{z}_{t-1}, \mathbf{z}_t)$, $\widehat{\mathbf{x}}_t^{(k)}$ and $\widehat{C}_t^{(k)}$.

4. If the absolute difference between the old and new log-likelihood is below a certain threshold, terminate the EM algorithm and output $\boldsymbol{\theta}$. Update the old log-likelihood otherwise.

[1] Christopher M. Bishop. *Pattern Recognition and Machine Learning (Information Science and Statistics)*. Springer, August 2006.

[2] Little, Roderick JA, and Donald B. Rubin. *Statistical analysis with missing data.* John Wiley & Sons, September 2002.
